# High impact psychiatric publishing – gender parity within reach?

**DOI:** 10.1192/j.eurpsy.2023.59

**Published:** 2023-07-19

**Authors:** A. Gmeiner

**Affiliations:** Social Psychiatry, Medical University of Vienna, Vienna, Austria

## Abstract

**Abstract:**

Andrea Gmeiner^1^, Melanie Trimmel^1^, Amy Gaglia^1,2^, Beate Schrank^3^, Stefanie Süßenbacher-Kessler ^1^, Michaela Amering^1^

^1^Division of Social Psychiatry, Department of Psychiatry and Psychotherapy, Medical University of Vienna, Austria ^2^Division of Psychology, Bangor University Wales,UK ^3^Department of Psychiatry, Karl Landsteiner University for Health Sciences, Austria

Gender parity, authorship, geographic and subject matter diversity are declared goals in the academic publishing world. Recent data on the progress towards these goals suggest that changes and a shift towards diversity have been happening over the last decades. Examples include significantly increasing numbers of female first and senior authors between 2008 and 2018 (Hart et al, 2019) over a wide range of journals. Our own data on trends in three high-impact psychiatric journals over a 25-year time period from 1994 until 2019 suggest that female first, female senior, and female overall authorship have increased significantly over the quarter of a century covered. Results do indicate that gender parity in first authorship was reached in the category of original research articles for the first time in 2019 (Gmeiner et al, 2022). However, data also showed the remaining underrepresentation of women in senior authorship positions in line with the *leaky pipeline* phenomenon. Gender differences in publication trends with regards to subject matters and topics in the 2004/14/19 part of this sample showed the percentage of female first authors exceeding 50% in the two most frequent subject matters ‘basic biological research’ and ‘psychosocial epidemiology’ in 2019 (Trimmel et al, submitted for publication). Although the percentage of female first authors in the three most common target populations under study (mood disorders, schizophrenia, general mental health) increased from 2004 to 2019, gender equality has not yet been achieved in these fields. Consistent monitoring of publication trends and gender distribution by researchers and academic journals needs to identify and counteract the areas of underrepresentation of women.

Hart, K. L., Frangou, S., & Perlis, R. H. (2019). Gender Trends in Authorship in Psychiatry Journals From 2008 to 2018. *Biological psychiatry*, *86*(8), 639–646.; Gmeiner A, Trimmel M, Gaglia-Essletzbichler A, Schrank B, Süßenbacher-Kessler S, Amering M, (2022). Diversity in high-impact psychiatric publishing: gender parity within reach? *Archives of women’s mental health*, *25*(2), 327–333.

**Disclosure of Interest:**

None Declared

